# Link Between Metabolic Syndrome, Blood Lipid Markers, Dietary Lipids, and Survival in Women with Early-Stage Breast Cancer

**DOI:** 10.3390/nu16213579

**Published:** 2024-10-22

**Authors:** Christine Bobin-Dubigeon, Loic Campion, Clémence Bossard, Elsa Rossignol, Jean-Sébastien Frenel, Mario Campone, Jean-Marie Bard

**Affiliations:** 1 Nantes Université CNRS, US2B, UMR 6286, 44000 Nantes, France; clemence.bossard@ico.unicancer.fr; 2 ICO René Gauducheau, Bd Jacques Monod, 44805 Nantes Saint Herblain CEDEX, France; loic.campion@ico.unicancer.fr (L.C.); elsa.rossignol@ico.unicancer.fr (E.R.); jean-sebastien.frenel@ico.unicancer.fr (J.-S.F.); mario.campone@ico.unicancer.fr (M.C.); 3 Centre de Recherche en Nutrition Humaine Ouest (CRNH), 44093 Nantes, France; jean-marie.bard@univ-nantes.fr

**Keywords:** breast cancer, nutrition, lipids, paraoxonase, metabolic syndrome, dietary habits

## Abstract

Background/Objectives: Nearly 10% of cancers could be prevented through dietary changes. In addition, breast cancer (BC) is the most common cancer in women worldwide. Inadequate diet may lead to several metabolic abnormalities, including metabolic syndrome (MS). The goal of our study is to evaluate the link between survival after BC and MS, as well as diet lipids and circulating lipids. Methods: This study was performed in an early-stage BC cohort (n = 73): MS, dietary lipids, and circulating biological parameters, including leucocyte expression in cholesterol carriers (ATP-binding cassette transporter ABCA1, ABCG1), were determined before any medication intervention. The data of each patient were analyzed using univariate logistic regression and are expressed by HR, 95%CI [5th–95th]. All these parameters were explored with survival parameters using Cox regression analyses. Results: Overall survival (OS) and invasive disease-free survival (iDFS) were significantly longer for the women without metabolic syndrome with HR 4.7 [1.11–19.92] and *p* = 0.036, and 3.58 [1.23–10.44] and *p* = 0.019, respectively. The expression of ABCG1 in peripheral leucocytes, an ATP-binding cassette transporter involved in cholesterol and phospholipid trafficking, is significantly associated with iDFS (1.38 [1.1–1.9], *p* = 0.0048). MS is associated with more pejorative survival parameters in early-stage breast cancer. Paraoxonase (or PON) activities differ according to PON gene polymorphism, but also diet. A link between PON activities and survival parameters was suggested and needs to be clarified. Conclusions: This study emphasizes the link between survival parameters of early-stage breast cancer, metabolic syndrome, and some parameters related to lipid metabolism.

## 1. Introduction

Breast cancer is the most common cancer in women worldwide with more than 2.26 millions new cases diagnosed in 2020 [1]. Diet, nutrition, and physical activity affect breast cancer risk, but also breast cancer survival and recurrence.

A diet rich in lipids may be a risk factor for many diseases, especially cardiovascular disease, but also for breast cancer [2]. However, the quality of the lipids must be taken into account. In fact, the Mediterranean diet, based on an abundant consumption of extra virgin olive oil and foods of plant origin in particular, has been suggested to have a protective effect against the occurrence of breast cancer [2], especially in menopausal women [3]. A poor lifestyle and an unbalanced and too rich a diet, associated with being overweight in postmenopausal women, are modifiable risk factors for BC, whereas being overweight seems to be associated with a lower risk in premenopausal women.

Metabolic syndrome (MS), which has been extensively studied in the cardiovascular context, is defined as a cluster of risk factors for cardiovascular disease (hyperglycemia, hypertriglyceridemia, low HDL cholesterol, visceral obesity, and high blood pressure), usually associated with hyperinsulinemia [4]. More recently, common mechanisms between cancer and metabolic syndrome, such as inflammation, insulin resistance, and excess adiposity, have prompted interest.

Dietary intake, especially lipid intake, may also play a role in various inflammatory processes [5], and the impact of food intake on inflammation could be determined by evaluating a dietary inflammation score through dietary questionnaire analyses [6].

Lipids play an important role in the development of tumors. Indeed, lipids are important for maintaining cellular homeostasis, but they are also used to synthesize cellular signaling molecules [7]. Among many lipid actors, we have focused for two decades on the expression of different genes such as LXRb- and LXR-dependent proteins such as ABCA1 and ABCG1 involved in cholesterol efflux from cells [8,9]. In previous studies, we have identified another actor of lipid metabolism, paraoxonase (PON), as a potential marker of survival in patients with breast cancer recurrence. Its role and purpose are not completely discovered; yet, it is commonly recognized that it plays an antioxidant role [10,11].

Moreover, its role in some diseases such as cardiovascular disease, inflammatory disease, and cancer has been shown in previous studies. Indeed, in the main cancer localizations, plasmatic PON activities are drastically lower in patients compared to controls [10]. Diet may influence PON, as high fat intake has been shown to be associated with lower activity of the enzyme [12], and it has also been shown that some genetic polymorphism (L55M and Q192R) can impact PON1 activity [13,14].

The goals of our study are to evaluate the link between metabolic syndrome, blood lipid markers, dietary lipids, and survival parameters in a cohort of patients suffering from an early-stage breast cancer positive for hormone receptors.

## 2. Materials and Methods

### 2.1. Patients

#### Study Design

Eligible patients of this multicenter open-label phase 2 trial, called TAM, were women aged 18 years or older, newly diagnosed for early-stage breast cancer (tumor size > 15 mm) with estrogen-receptor-positive, HER2-negative operable primary cancer, without evidence of metastatic spread. This study was carried out in accordance with the guidelines for Good Clinical Practice and the Declaration of Helsinki and was approved by the CPP Ouest IV (EUDRACT 2008-007652-10). Written informed consent and specific genetic consent were obtained from each participant. The current study protocol is available online (https://clinicaltrials.gov/ct2/show/NCT01220076, accessed on 6 July 2024). Tamoxifen 20 mg per day was prescribed for 5 weeks after enrollment, prior to breast surgery.

One of the secondary objectives was to examine the interaction of diet and tamoxifen treatment. Nutritional parameters such as dietary habits and circulating nutritional biomarkers were evaluated at baseline, immediately after inclusion and before any treatment.

**Figure 1:**
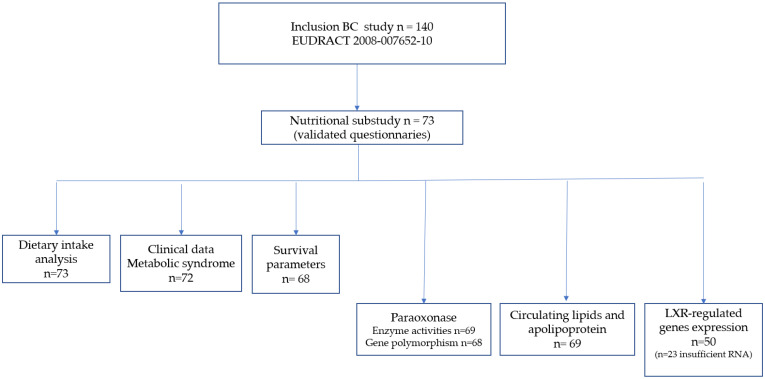
Flowchart of this study.

The flowchart of this study is summarized in Figure 1.

### 2.2. Metabolic Syndrome

Metabolic syndrome (MS) was defined for women according to [15] which requires the presence of three of the five following criteria: (1) hypertriglyceridemia (~1.50 g/litre or 1.7 mmol/litre); (2) high arterial blood pressure (~130/85 mm Hg); (3) low HDL cholesterol (<0.5 g/L or 1.1 mmol/L); (4) hyperglycemia (~1.0 g/L or 5.6 mmol/L); and (5) waist measurement equal to or higher than 88 cm. Treatment for hypertension, hyperglycemia, or dyslipidemia was considered a positive criterion.

For each patient, the clinico-biological data (age, including menopausal status, weight, height, breast cancer subtype, tumor overexpression) were collected at baseline, prior to any treatment, to assess the prevalence of MS. Performance status (PS, a standard criterion for measuring the impact of the disease on a patient’s daily living abilities) was collected for each patient. Overall survival (OS), invasive disease-free survival (iDFS), and relapse-free survival (RFS) were explored in relation to MS.

### 2.3. Dietary Lipids and Lipidic Biomarkers

#### 2.3.1. Dietary Intake

Dietary intake was assessed at baseline using 3-day self-report questionnaires (2 weekdays and 1 weekend day) to evaluate the relationship between diet, metabolic syndrome, and lipid biomarkers as described [16]. This 72 h food recall provides detailed information on the quality and quantity of the meals consumed. For each patient, dietary intakes were converted into energy and macro- and micronutrients according to the Ciqual French food composition table 2020 [17] by using Nutrilog software (version 3.20). Validation of dietary intake was performed according to the Golberg cut-off for energy intake (>1.35) as described in [18].

A dietary inflammatory score (DII score) was calculated for each validated questionnaire according to Shivappa’s adapted method [6,19,20,21]. The DII score was calculated by taking into account the intake of 27 nutriments (total energy, alcohol, vitamins B6 and B12, beta carotene, carbohydrates, cholesterol, fat, fiber, folic acid, iron, magnesium, monounsaturated fatty acids (MUFAs), polyunsaturated fatty acids (PUFAs), niacin, protein, omega 3 and omega 6 fatty acids, riboflavin, saturated fatty acids, selenium, thiamin, vitamins A, C, D, and E, and zinc). The DII score was explored in relation to the survival parameters and the biological parameters related to paraoxonase activities.

#### 2.3.2. Circulating Lipids and Apolipoproteins

Total cholesterol, plasma triglycerides, high-density lipoprotein cholesterol (HDL-C), and low-density lipoprotein cholesterol (LDL-C) concentrations were measured at baseline using enzymatic kits from Diasys^®^, according to the manufacturer’s instructions (Grabels, France). Apolipoproteins were quantified by LC MS/MS as previously described [22].

#### 2.3.3. LXR-Regulated Genes of Cholesterol Trafficking

In order to explore the LXR-regulated genes of cholesterol trafficking and lipidic patterns, nucleic acids were extracted from Tempus blood RNA tubes (Thermo Fisher, Nantes, France) at baseline and at week 5, as previously described [23]. Relative quantification was performed using the ΔCT method. The genes involved in cholesterol trafficking and studied in this work were ABCA1, ABCG1, PON2, and LXRβ. The genes and Taqman^®^ probes (Life Technologies, Villebon sur Yvette, France) were as follows (gene name, assay ID): ATP-binding cassette, sub-family A, member 1, Hs00194045_m1 for ABCA1; ATP-binding cassette, subfamily G (WHITE), member 1 (ABCG1), Hs01555189_m1 for ABCG1; nuclear receptor subfamily 1, group H, member 2 (NR1H2), Hs01027208_m1 for LXRβ; and paraoxonase, Hs00165563_m1 for PON2, as described in [23]. The values were normalized against three housekeeping genes as described previously.

The expression of these genes was explored in relation to the three survival parameters and to the intake of dietary fatty acids.

#### 2.3.4. Paraoxonase

At baseline, serum paraoxonase (PON1) activities were quantified on three different substrates, paraoxon (PON), lactonase (LAC), and phenylacetate (ARE), as adapted from a previous description [24]. Hydrolysis rate was followed in duplicate using a continuously recording spectrophotometer (TECAN, Männedorf, Switzerland, Infinite M200).

In addition, the genotypic frequencies of PON1 L55M and Q192R were determined by an allelic discrimination assay using real-time PCR using TaqMan fluorogenic probes as described in [25], on saliva samples (FTA cards). Biological parameters related to paraoxonase activities were also calculated such as individual enzyme activities/HDL ratio and enzyme activities/apolipoprotein A1 ratio as previously described [22,26].

### 2.4. Statistical Considerations

Qualitative factors are described by means of the frequency of their respective modalities and compared using Pearson’s Chi-square test (or Fisher’s test). Continuous factors are described by means of their median (IQR) and compared using the Mann–Whitney test (or Kruskal–Wallis test if more than 2 groups).

Invasive disease-free survival (iDFS) was defined as the time from the date of inclusion in the study to the date the event occurred first (as defined in the DATECAN guidelines) or censored at the last event-free date; iDFS was calculated and plotted using Kaplan–Meier curves. Survival curves were compared using the log-rank test. Overall survival (OS) was defined as the time from the date of inclusion to the date of death.

The median of follow-up was calculated using the inverse Kaplan–Meier method.

Analyses were performed using StataSE 17.0 software (StataCorp, College Station, TX, USA). The tests were performed in two-tailed formulation and the significance limit was set at 5%. Multivariate analyses were performed by including MS status, age, and Dietary Inflammatory Index with survival parameters.

## 3. Results

### 3.1. Description of the Study Cohort and Metabolic Syndrome

The study cohort included n = 73 patients, and the clinico-biological parameters are synthesized in Table 1 (one missing datum). The prevalence of MS in the whole study population was 22/72 (30.5%) according to the NCEP definition.

As expected, SM+ patients were significantly older, mainly with menopausal status with a lower performance status. In the same way, the subtype of breast cancer differed according to the SM status.

**Figure 2:**
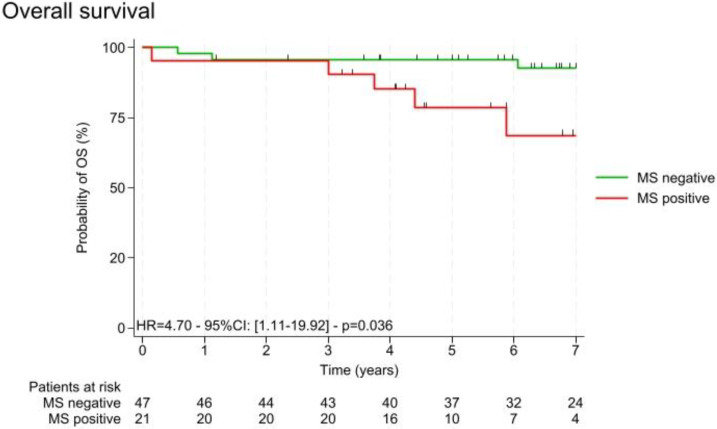
Overall survival with and without the presence of metabolic syndrome (MS+ vs. MS−); n = 68.

We explored the interaction between metabolic syndrome and survival parameters. As shown in Figure 2, overall survival (OS) was significantly longer in women without metabolic syndrome (MS–) with HR 4.7 [1.11–19.92], *p* = 0.036.

**Figure 3:**
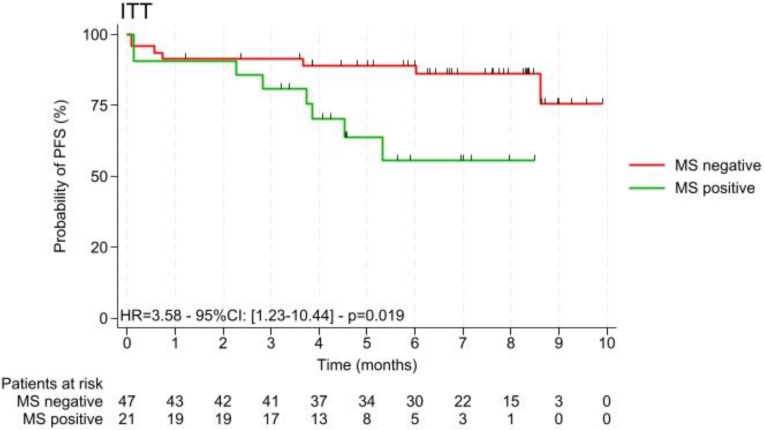
iDFS with and without the presence of metabolic syndrome (MS+ vs. MS−); n = 68.

In the same way (Figure 3), iDFS was significantly longer in women with MS– with HR 3.58 [1.23–10.44], *p* = 0.019, although the metabolic syndrome status did not appear to have influence on the relapse-free survival (HR = 2.14 [0.57–8.08], *p* = 0.262).

However, on multivariate analysis including age, MS still remained significantly associated with iDFS but not with OS.

### 3.2. Dietary Habits and Dietary Inflammatory Index

In patients with validated dietary questionnaires (n = 73), the median [25th–75th] daily energy intake and macronutrients carbohydrate, protein, and lipids were 2042 kcal/d [1550–2910], 220 g/d [188–254] (43.4% of energy intake or EI), 85.9 g/d [76.9–100] (16.7% EI), and 82.9 g/d [72.3–92] (36.7% EI), respectively. More specifically for lipids, the daily intakes of total FA, saturated FAs, MUFAs, and PUFAs ω3 and w6 daily amounts were 61.4 g/d [51.1–76.0], 30.3 g/d [20.8–37.4], 22.7 g/d [17.5–27.4], 8.8 g/d [6.6–13.1] g/d, 1.1 g/d [0.9–1.9], and 7.2 g/d [5.2–9.9], respectively.

For each validated questionnaire, the Dietary Inflammatory Index or DII was calculated by including 27 items as described above. The median value of the DII was −0.43 [25th–75th: −1.61, 0.26]. The negative value of the DII score suggests that the dietary habits in our cohort tend to be anti-inflammatory. None of the survival parameters, such as overall survival, relapse-free survival, and invasive disease-free survival, were associated with DII score with HR [95% CI]: 1.43 [0.92–2.23] (*p* = 0.11), 0.996 [0.69–1.43] (*p* = 0.98), and 1.11 [0.82–1.50] (*p* = 0.5), respectively. Moreover, the DII score was not associated with any other biological parameters related to paraoxonase activities.

### 3.3. LXR-Regulated Genes of Cholesterol Trafficking, Lipidic Dietary Habits, and Survival Parameters

The expression of different LXR-regulated genes of cholesterol trafficking in peripheral leukocytes was evaluated at baseline and at week 5 (Table 2).

The expression of LXR-regulated genes of cholesterol trafficking in peripheral leukocytes remained stable after tamoxifen treatment, as no difference was observed between baseline and week 5.

The link between the expressions of these genes at baseline and dietary habits, more specifically lipids, was explored, in addition to the relation with the survival parameters. The expression of ABCG1 at baseline, an ATP-binding cassette transporter involved in cholesterol and phospholipid trafficking, was significantly associated with invasive disease-free survival (1.38-[1.1–1.9], *p* = 0.048) and tended to be significant with overall survival (1.44-[1.0–2.1], *p* = 0.055). In addition, ABCA1, PON2, and LXRb expression in leukocytes were associated with overall survival with HR [95%CI]: 1.38 [0.56–3.39], *p* = 0.48; 0.53 [0.02–19.06], *p* = 0.73; and 1.28 [0.65–2.53], *p* = 0.47, respectively. Moreover, no link was observed with invasive disease-free survival.

**Figure 4:**
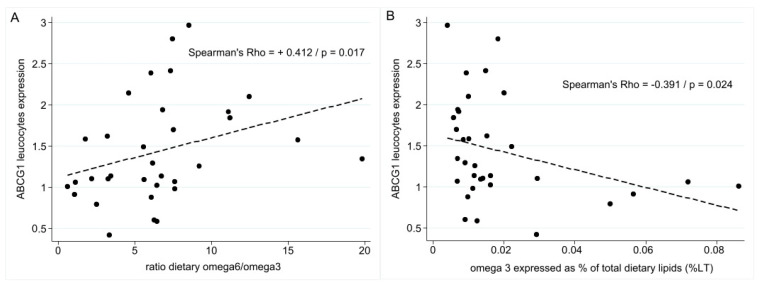
Relationship between ABCG1 leucocyte expression and the ratio of dietary ω6/ω3 (**A**) and omega 3, expressed as % of total dietary lipids (**B**).

As described in Figure 4A, high ABCG1 expression in peripheral leukocytes is correlated with a higher ratio of dietary PUFA ω6/ω3 (Spearman r correlation = 0.412, *p* = 0.01) and % of ω3 expressed as % total dietary lipids (B) (Spearman r correlation = −0.391, *p* = 0.024).

### 3.4. Paraoxonase

#### 3.4.1. Link Between Enzyme Activities and Paraoxonase Genotypes

The three enzymatic activities of paraoxonase ARE, LAC, and PON quantified at baseline on the cohort studied were 21.6 mmol/L/min [17.1–31.9], 0.174 µmol/L/min [0.155–0.192], and 114 µmol/L/min [64–201], respectively. As shown in Table 3, the genetic polymorphism L155M did not seem to impact any of the three enzyme activities PON, ARE, or LAC (n = 69).

Conversely, ARE, PON, and LAC activities differed according to the Q192R polymorphism (Table 4). Indeed, the presence of the allele R seemed to decrease the ARE activity, whereas increased LAC and PON activities were observed (n = 68).

#### 3.4.2. Link Between Paraoxonase Activities and Lipid Dietary Habits

The ARE activity is positively correlated with the daily dietary PUFA ratio ω6/ω3 with R = 0.265 (*p* = 0.027). The daily amount of dietary PUFA ω6 is also positively associated with LAC activity and the ARE activity/apolipoprotein A1 ratio with R = 0.24 (*p* = 0.04) and R = 0.278 (*p* = 0.036), respectively. No other correlation was observed between paraoxonase activities expressed as HDL or the apolipoprotein ratio.

#### 3.4.3. Link Between Paraoxonase Activities and Survival Parameters

Paraoxonase activities, whatever their expression—global activities, the ratio on HDL, or the ratio on apolipoprotein A1—were not associated with overall survival in breast cancer patients. However, our results suggest that a higher LAC/HDL ratio was associated with an increased risk of recurrence (HR = 2.54 [1.25–5.19], *p* = 0.01). In addition, the PON/apoA1 ratio was inversely associated with iDFS (HR = 0.34 [0.12–0.98], *p* = 0.04).

## 4. Discussion

The aim was to explore the link between survival metabolic syndrome, lipid biomarkers, and dietary habits with survival parameters in a cohort of women with early-stage hormone-dependent breast cancer (BC).

The molecular subtype of breast cancer is luminal A, which is the main type of early-stage BC. The clinical and anthropometric characteristics are in agreement with the usually described data.

Among the different objectives of this work, we aimed to determine the frequency of metabolic syndrome (MS) in the studied population and explore its link with survival parameters. A cluster of metabolic risk factors including abdominal obesity, hypertension, dyslipidemia, insulin resistance, and low HDL-C plasma concentration defines metabolic syndrome [15]. For many decades, this syndrome has been associated with an increased cardiovascular risk [27]. This is mainly due to the poor quality of nutritional habits with high saturated fatty acids and carbohydrates [28,29]. The quality of dietary fatty acids must be taken into account as a Mediterranean diet regimen rich in olive oil intake clearly improved MS criteria [29]. The prevalence of MS reaches epidemic proportions in Western countries and varies according to sociodemographic status, age, and gender. The prevalence of MS in our cohort (30.5%) is consistent with that described in epidemiological studies such as MONICA related to the age of patients (26% for 55–65-year-olds [30]) and in women with breast cancer [31], and will probably increase in the next decade in parallel with obesity. The link between MS and cancer has been explored with a central role in insulin resistance and insulin-like GF1, but also secreted adipokines and free fatty acids [32].

Therefore, MS is strongly suggested to increase the risk of several cancer localizations such as breast cancer as described in a meta-analysis [33] in postmenopausal women (1.56, *p* = 0.017).

Our results show a link between metabolic syndrome and the decrease in overall survival, and also iDFS. These data are in complete agreement with those previously published [34,35,36,37], for example, in the large epidemiologic study WHI, which concluded that postmenopausal women with 3–4 MS components had a higher risk of death from breast cancer [36] (compared to none).

Recent work in early ER+ cancer [38] even suggests that the relative risk of endocrine-resistant tumor was 1.4-fold greater for patients with MS (*p* = 0.0197).

The dietary habits of the study population were explored by self-assessment questionnaires. Different strategies could be used to evaluate dietary patterns of a population, such as a food frequency questionnaire, a food diary or food record with data collected for at least 2 days, or two or more 24 h recalls. Nevertheless, a standardized approach could be helpful [39].

The macronutrients and lipid consumption of our cohort are in the same range as those described in a large French nutritional epidemiological study INCA3 [40] for the main parameters (energy, lipids, carbohydrates, total protein, and % energy amount). It seems that our patients’ dietary habits are closer to the French dietary recommendations with, for example, a daily energy intake around 2072 kcal/d vs. 1787 kcal/d for women age-matched to INCA3 for a recommendation of 1800–2200 kcal/d. Our patients also have the same dietary intakes of total FA and PUFAs ω6 and ω3 compared to INCA3. However, our approach to evaluate dietary intakes in our cohort could be improved by using a more complete nutrient database than Ciqual 2020, such as EPIC nutrient database [41].

The impact of food intake on inflammation could be determined by the dietary inflammatory score [8]. The dietary inflammatory score is described as a new tool that enables researchers to describe the pattern of people’s diet to determine whether their food intake is more anti- or pro-inflammatory. The interest in this score has risen for two decades and originally included 45 nutrients to characterize the inflammatory profile of the diet [6].

The link between breast cancer risk and DII has been largely suggested in Swedish, Chinese, and Iranian studies and meta-analyses [20,42,43,44]. Few studies like ours have investigated the link between DII score and breast cancer survival parameters, and like Zuchetto et al. (2017) [45], our findings did not suggest an association between the inflammatory potential of the diet, measured by the DII, and breast cancer survival parameters, in contrast to [46] where a higher risk of death from BC was associated with the consumption of a more pro-inflammatory diet at baseline (HR_Q5*vs*Q1_, 1.33; 95% CI, 1.01–1.76; *P*_trend_ = 0.03). Our results could probably be explained by the small number of studied questionnaires but also by the inability to include different components (like in the original DII calculation), such as ginger, turmeric, garlic, oregano, pepper, rosemary, eugenol, saffron, flavan-3-ol, flavones, flavonols, flavonones, and anthocyanidins. Experiments have been undertaken to validate our DII as described in [47].

To explore the impact of the cell lipidic metabolic actors on breast cancer survival parameters, LXR-regulated genes of cholesterol trafficking expressions in peripheral leukocytes were evaluated, at baseline.

The link between the peripheral expression of these genes and survival parameters has not been extensively studied. In the present study, ABCG1 (involved in cholesterol efflux from the cell) expression at baseline was significantly associated with iDFS and tended to be associated with overall survival.

On the other hand, in a previous work, we had shown that tumor ABCG1 was related to the disease severity with a lower expression in the highest SBR-grade tumor (pejorative situation) [23]. The tumor expression levels of LXR-regulated genes of cholesterol trafficking also differ according to the subtype of metastatic breast cancer as previously suggested [48].

The expression of liver X receptor (LXR)-regulated genes of cholesterol trafficking such as ABCG1 expression in cholesterol influx and efflux mediators is also influenced by dietary intake. Our data suggest that ABCG1 expression in PBMC is associated with a high dietary PUFA ratio ω6/ω3, which is not really in agreement with the literature. According to the literature, the expressions of ABCG1 and ABCA1 on PBMC are increased with high intakes of SFA rather than PUFA [48].

However, the significance of our results is limited by the small number of patients and therefore needs to be confirmed by a larger-cohort approach.

Previously extensively studied from a cardiovascular point of view, paraoxonase 1 is carried in the plasma by high-density lipoproteins (HDLs) and protects low-density lipoproteins from oxidation (LDL). It is well accepted that PON activity is lower in cancer patients than in controls and, in a previous work, we identified paraoxonase as a potential marker of survival in patients with breast cancer recurrence [49]. The enzymatic activities quantified in our cohort are consistent with those previously described by our team. However, it seems difficult to compare our results with other published data as many different methods of enzymatic measurement have been described [50,51]. As it has been described, PON1 levels depend on genetic polymorphisms [52]. In our cohort, the frequencies of the polymorphisms PON1_192_ and PON1_55_ are in agreement with those described in the Caucasian population (recent review in [53]).

PON1 activities are modified by dietary intake. The consumption of polyphenols-enriched diets may increase PON1 activity. The Mediterranean diet has also been shown to have a beneficial effect on PON1 activity. Extra virgin olive oil (ω9) seems to increase PON1 activity through the oleic acid enrichment of phospholipids present in HDL. This oil favored PON1 activity and increased hepatic PON1 mRNA and protein expression. These effects could be attributed to minor components present in this oil (terpenes, phytosterols). The high consumption of fruits and vegetables in the Mediterranean diet could also contribute to the modulation of paraoxonase activities [54].

In our previous study, paraoxonase 1 was identified as a marker of short-term death with cancer recurrence [23]. This new study confirms the interest of the early quantification of paraoxonase activity as we have shown the significant inverse association between PON/apoA1 and iDFS, but also a positive association between LAC/HDL and RFS.

These unexpected data need to be confirmed in a large breast cancer cohort.

## 5. Conclusions

MS is associated with reduced survival parameters in early-stage breast cancer. The peripheral leukocytes expression of ABCG1 involved in cholesterol trafficking is related to survival parameters. Despite the smaller sample size of the present study, our results are in agreement with previously published data on the impact of genetic and non-genetic factors (diet) to modulate paraoxonase activities. The impact of PON1 on iDFS in women with early-stage breast cancer is described for the first time. A mechanistic approach and larger clinical studies are needed to confirm these data.

This study highlights the relationship between early-stage breast cancer survival parameters, metabolic syndrome, and some parameters related to lipid metabolism.

## Figures and Tables

**Table 1:** Clinico-biological parameters of the studied population associated with validated dietary questionnaires.

	SM+ (n = 22)	SM− (n = 50)	*p*-Value
Age (years) *	65 (54–75)	52 (44–63)	0.006
BMI (kg m^−2^) *	28.5 (25.7–30.9)	22.5 (20.6–24.9)	<0.001
Menopausal status **	18 (81.8%)	23 (46%)	0.005
Performance status = 0	17 (77.3%)	50 (100%)	0.002
Type of cancer **			0.03
Invasive carcinoma of no special type (ductal)	13 (59.1%)	41 (82.0%)	
Invasive lobular carcinoma	9 (40.9%)	7 (14.0%)	
Other	0 (0%)	2 (4.0%)	
Overexpression of estrogen receptor	22 (100%)	50 (100%)	
HER2+	0%	0%	
Circulating lipids (mmol/L)			
Plasma cholesterol	5.42 (4.74–5.94)	5.68 (4.94–6.67)	0.144
Plasma triglycerides	1.07 (0.74–1.59)	0.94 (0.72–1.30)	0.309
HDL cholesterol	1.26 (1.15–1.51)	1.81 (1.58–2.03)	<0.001
LDL cholesterol	3.26 (2.73–3.86)	3.42 (2.91–4.21)	0.303

* Median (25th–75th); ** frequency expressed as *n* (percentage); Mann–Whitney test; n = 72; n = 1 missing datum.

**Table 2:** LXR-regulated genes of cholesterol trafficking expression of peripheral leucocytes at baseline and week 5 (arbitrary units = AU).

Genes	AU (n = 50)		
	Baseline	Week 5	*p*-Value
ABCG1	1.47 [1.06–1.85]	1.23 [0.94–1.70]	0.72
ABCA1	1.40 [1.01–2.10]	1.40 [1.01–2.10]	0.61
PON2	0.58 [0.50–0.73]	0.65 [0.52–0.70]	0.47
LXRb	1.46 [0.95–2.34]	1.49 [1.01–2.30]	0.98

Results expressed as median [25th–75th]; missing data n = 23 insufficient RNA.

**Table 3:** Paraoxonase activities according to the PON L155M gene polymorphism.

Activities	L/L (n = 30)	M/L (n = 34)	M/M (n = 5)	P Global	LL vs. ML	P ML vs. MM	LL vs. MM
ARE mmol/L/min	20.5 [17.4–25.9]	23.9 [15.9–38.2]	50.9 [18.2–59.0]	0.18	ns	ns	ns
LAC µmol/L/min	0.177 [0.16–0.187]	0.174 [0.15–0.20]	0.171 [0.13–0.177]	0.71	ns	ns	ns
PON µmol/L/min	109 [64–165]	123.5 [71–209]	81 [51–112]	0.34	ns	ns	ns

Results expressed as median [25th–75th], n = 69 patients; missing data n = 4.

**Table 4:** Paraoxonase activities according to PON Q192R gene polymorphism.

Activities	Q/Q	R/Q	R/R	P			
	(n = 34)	(n = 25)	(n = 9)	P Global	QQ vs. RQ	RQ vs. RR	QQ vs. RR
ARE mmol/L/min	27.9 [18.5–38.0]	19.3 [14.8–23.9]	18.2 [14.8–31]	0.036	0.016	1	0.11
LAC µmol/L/min	0.172 [0.143–0.18]	0.174 [0.16–0.20]	0.187 [0.18–0.21]	0.032	0.27	0.081	0.01
PON µmol/L/min	69 [52.2–99.5]	168 [120–218]	242 [69–285]	<0.0001	<0.0001	0.61	0.04

Results expressed as median [25th–75th], n = 68 patients; missing data n = 5.

## Data Availability

The original contributions presented in the study are included in the article, further inquiries can be directed to the corresponding author.
